# Changes in pain and insulin-like growth factor 1 in fibromyalgia during exercise: the involvement of cerebrospinal inflammatory factors and neuropeptides

**DOI:** 10.1186/ar3902

**Published:** 2012-07-09

**Authors:** Jan L Bjersing, Mats Dehlin, Malin Erlandsson, Maria I Bokarewa, Kaisa Mannerkorpi

**Affiliations:** 1 Department of Rheumatology and Inflammation Research, Institute of Medicine, Sahlgrenska Academy, University of Gothenburg, Guldhedsgatan 10, Box 480, 40530 Göteborg, Sweden; 2 Sahlgrenska University Hospital, Rheumatology, Gröna stråket 14, 413 45 Göteborg, Sweden; 3 Sahlgrenska University Hospital, Physiotherapy and Occupational therapy, Vita stråket 13, 413 45 Göteborg, Sweden

## Abstract

**Introduction:**

Fibromyalgia (FM) is characterized by chronic pain. Impaired growth hormone responses and reduced serum insulin-like growth factor 1 (IGF-1) are common in FM. The aim was to examine changes in serum IGF-1, cerebrospinal fluid (CSF), neuropeptides, and cytokines during aerobic exercise in FM patients.

**Methods:**

In total, 49 patients (median age, 52 years) with FM were included in the study. They were randomized to either the moderate- to high-intensity Nordic Walking (NW) program (*n *= 26) or the supervised low-intensity walking (LIW) program (*n *= 23). Patients participated in blood tests before and after 15 weeks of aerobic exercise. Changes in serum levels of free IGF-1, pain rating on a 0- to 100-mm scale, pain threshold, and 6-minute walk test (6MWT) were examined. CSF, neuropeptides, matrix metalloproteinase 3 (MMP-3), and inflammatory cytokines were determined. Nonparametric tests were used for group comparisons and correlation analyses.

**Results:**

Serum free IGF-1 levels did not change during 15 weeks of exercise between the two groups, although the 6MWT significantly improved in the NW group (*p *= 0.033) when compared with LIW. Pain did not significantly change in any of the groups, but tended to decrease (*p *= 0.052) over time in the total group. A tendency toward a correlation was noted between baseline IGF-1 and a decrease of pain in response to exercise (*r *= 0.278; *p *= 0.059). When adjusted for age, this tendency disappeared. The change in serum free IGF-1 correlated positively with an alteration in CSF substance P (SP) levels (*r*^*s *^= 0.495; *p *= 0.072), neuropeptide Y (NPY) (*r^s ^*= 0.802; *p *= 0.001), and pain threshold (*r^s ^*= 0.276; *p *= 0.058). Differing CSF SP levels correlated positively to a change in pain threshold (*r*^*s *^= 0.600; *p *= 0.023), whereas the shift in CSF MMP-3 inversely correlated with an altered pain threshold (*r*^*s *^= -0.569; *p *= 0.034).

**Conclusions:**

The baseline level of serum free IGF-1 did not change during high or low intensity of aerobic exercise. Changes in IGF-1 correlated positively with a variation in CSF SP, NPY, and pain threshold. These data indicate a beneficial role of IGF-1 during exercise in FM.

**Trial registration**: ClinicalTrials.gov NCT00643006.

## Introduction

Fibromyalgia (FM) is characterized by chronic pain, tenderness [1], and enhanced central sensitivity [2]. Substance P (SP) is associated with central pain sensitivity in FM patients. Low-grade inflammation could be involved in the pathogenesis. Despite increased central sensitivity, long-term physical exercise appears to improve physical capacity and pain in FM, although not in all patients [3]. Exercise at moderate-to-high intensity results in better improvement of physical functions than does exercise at low intensity [4]. Some exercise studies have reported decreased pain after exercise intervention [3]. The biologic mechanisms controlling changes in pain in FM requires further elucidation.

Approximately one third of FM patients are estimated to suffer from growth hormone (GH) deficiency, with impaired growth hormone responses leading to reduced insulin-like growth factor 1 (IGF-1), a critical mediator of growth hormone [5-7]. A previous study found that GH dysfunction was associated with increased pain scores during an exercise test as well as with higher pre-exercise levels of interleukin-6 (IL-6) and interleukin-8 (IL-8) [8]. Regular exercise is expected to increase the resting level of IGF-1 in healthy individuals [9], but no increase was found in patients with FM who exercised for 6 months [10]. Together with insulin, IGF-1 is a central regulator for cell growth, survival, and energy metabolism in the body and the central nervous system (CNS). The major IGF-binding protein in serum and extracellular fluid is insulin-like growth binding protein 3 (IGFBP3). IGFBP3 did not change significantly in FM patients after 6 months of exercise [10].

Nerve growth factor (NGF) is increased in cerebrospinal fluid (CSF) in FM patients [11]. NGF supports neuronal growth, differentiation, and modulates neuroplasticity [12]. NGF promotes sensory nerve sprouting, SP release [13,14], and is induced by proinflammatory cytokines [15].

SP is increased in CSF of FM patients [16-18]. CSF SP levels are stable over time in FM patients; however, increases in SP correlates to small increases in pain [16].

Neuropeptide Y (NPY) serum levels are increased in FM patients but not associated with clinical severity [19,20]. NPY is neuroprotective [21,22], and counteracts inflammatory and neuropathic pain through the endogeneous opioid system [23,24]. NPY expression in ganglionic neurons is promoted by NGF [25].

Interleukin-6 (IL-6) is a proinflammatory cytokine and can induce short-term nociceptor hypersensitivity [26]. CSF IL-6 is increased in the complex regional pain syndrome [27] and radiculopathy [28], and serum IL-6 is increased in FM [29]. Interleukin-8 (IL-8) is also proinflammatory, is involved in persistent [29] nociceptor hypersensitivity, and is increased in chronic orthopedic pain [30]. IL-8 has been shown to be elevated in CSF [31] and in serum [29,32] in FM. Matrix metalloproteinase-3s (MMP-3s) are changed during exercise [33]. MMP-3 and other MMPs cooperate in remodeling of the extracellular matrix, in inflammatory processes [34], and in neural remodeling in the CNS [35,36].

The aim of the study was to study the effect of 15-week moderate- to high-intensity aerobic exercise on the level of serum bioactive IGF-1 in women with FM. Low-intensity aerobic exercise was used by a control group. Changes in IGF-1, IGFBP3, pain, and CNS markers of inflammation and neuropeptides were studied to get a better insight into the biologic mechanisms involved in pain in FM.

## Materials and methods

### Study design and subjects

Forty-nine patients with FM formed the study population. Patients were randomized either to a moderate- to high-intensity Nordic Walking (NW) program (*n *= 26) or a supervised low-intensity walking (LIW) program (*n *= 23). The supervised exercise programs were conducted twice a week for 40 to 45 minutes for 15 weeks. They participated in a blood test at baseline, after 15 weeks of exercise, and at 30 weeks of follow-up. The effects of Nordic walking on body function were reported previously [4], showing that NW resulted in better improvement in the 6-minute walk test (6MWT) and aerobic capacity, when compared with patients in LIW. Pain did not significantly change in any of the exercise groups. For patient characteristics, see Table 1.

**Table 1:** Patient characteristics and pharmacologic treatment

Patient characteristics (*n *= 49)	Median (interquartile range)
Age, years	52 (48-56)

Symptom duration, years	11 (7-15)

Tender points, *n *	15 (13-16)

**Pharmacologic treatment**	***n *(%)**

Analgesics, yes	40 (82%)

Antidepressants/sedatives, yes	31 (63%)

Criteria for inclusion: Women with FM, aged 20 to 60 years, and with interest in exercising outdoors twice a week for 15 weeks, to participate at blood test at baseline and after the exercise period. To ensure that the patients would manage the planned aerobic exercise, a bicycle test at 50 watts was included in the inclusion criteria. All included patients managed to perform the test. They were also invited to participate in an examination of cerebrospinal fluid, which, however, was not a criterion of inclusion. FM was defined by the ACR 1990 criteria [1]: a history of long-lasting generalized pain and pain in at least 11 of 18 tender points examined by manual palpation.

Criteria for exclusion: Patients not speaking or reading Swedish, other severe somatic or psychiatric disease, ongoing or planned physical therapy, including exercise, and inability to accept times for planned exercise sessions.

### Clinical measurements

The pain threshold was examined by using an algometer (Somedic Production AB, Sollentuna, Sweden), measured in kilopascals (kPa) [37]. The pain threshold was measured in two tender-point locations in the upper and lower extremities, respectively. The mean value was applied, and a higher value indicates better health.

Pain was rated on a visual analog scale (0 to 100) of the Fibromyalgia Impact Questionnaire (FIQ) [38] and reflects subjective estimation of overall pain intensity during the last week. A higher score indicates more-severe pain.

The 6-minute walk test (6MWT) reflects physical restriction related to the pain disorder. The patient was instructed to walk as quickly as possible but not to run. The distance covered was measured in meters [39].

### Blood and cerebrospinal fluid (CSF) sampling

Serum was collected at rest (*n *= 49) at baseline and after 15 weeks in the exercise program and at 30 weeks of follow-up (*n *= 40). Serum samples were acquired by venipuncture of the cubital vein.

Twenty-six patients agreed to participate in an examination of cerebrospinal fluid (CSF) at baseline, and 15 patients also participated after 15 weeks of exercise. CSF was collected through lumbar puncture through the L3/L4 interspace. Collected blood and cerebrospinal fluid samples were centrifuged at 800 *g *for 3 minutes, aliquoted, and stored frozen at -70°C until use.

### Laboratory analyses

Biologic markers were analyzed with enzyme-linked immunosorbent assay (ELISA) by using commercially available kits. Assays specific for IL-8 (DY208, detection limit 31 pg/ml), MMP-3 (DY513, 1.4 pg/ml), serum free bioactive IGF-1 (DY291, 4 pg/ml), and IGFBP3 (DY675, 0.125 ng/ml) were purchased from R and D Systems (Minneapolis, MN, USA). An assay specific for human IL-6 (M1916, 0.6 pg/ml) was purchased from Sanquin Reagents (Amsterdam, The Netherlands). Assays specific for SP (FEK-061-05, 1 pg/ml) and NPY (FEK-049-03, 1 pg/ml) were purchased from Phoenix Pharmaceuticals (Burlingame, CA, USA). The human NGF-specific assay was purchased from Promega (Madison, WI, USA; 4 pg/ml). All assays were run according to recommendations of the manufacturers. Ordinary colorimetric ELISA was read with a Spectramax 340 from Molecular Devices (Sunnyvale, CA, USA), and fluorescent ELISA assays were read with a Mithras LB940 from Berthold Technologies (Bad Wildbad, Germany).

### Ethics

The study was approved by the ethics committee of Sahlgrenska University Hospital (220-09). Written and verbal information was given to all patients, and written consent was obtained from all patients.

### Trial registration

ClinicalTrials.gov NCT00643006.

### Statistics

Descriptive data are presented as median, interquartile range, the number, and percentage. The Mann-Whitney *U *test and Kruskal-Wallis test were used to analyze the differences in continuous variables between the groups. The mean of baseline and posttest values was calculated for each individual, and these means were used in group comparisons. The Wilcoxon signed-rank test was used for comparisons of continuous variables within groups. Relations between the variables were examined with the Spearman correlation coefficient. Partial correlational analyses were performed to adjust for baseline pain and age. To control for possible Type I errors, the upper limit of number of false significances was calculated by the following formula: (number of tests - number of significant tests on the level of α) × α/(1-α). All significant tests were two-tailed, and values of *p *< 0.05 were considered significant. All statistical evaluation of data was done by using the statistic program SPSS Statistics v. 19.

## Results

### Clinical effects of exercise

Baseline values for 6MWT, pain, and pain threshold are presented in Table 2. The 6MWT was found significantly to improve (*P *= 0.033) in the NW group (median increase, 36.5 m) when compared with the LIW (median increase, 8.0 m). Changes in pain levels and pain threshold did not differ significantly between groups (Table 2).

**Table 2:** Clinical data, IGF-1, and IGFB-3 for Nordic walking group and low-intensity walking group

	Nordic walking			Low-intensity walking			Difference in change between groups at at 15 weeks	Difference in change between groups at at 30 weeks
			
	Baseline	15 weeks to 0 weeks	30 weeks to 0 weeks	Baseline	15 weeks to 0 weeks	30 weeks to 0 weeks		
	
	Median (range)	Diff median (range) *P *value^a^	Diff median (range) *P *value^a^	Median (range)	Diff median (range) *P *value^a^	Diff median (range) *P *value^a^	*P *value^b^	*P *value^b^
Pain threshold kPa	199.2 (106.2 to 372.3)	-6.6 (-84.6 to 75.6) *P *= 0.454	13.9 (-109.2 to 94.1) *P *= 0.278	215.4 (105.9 to 380.5)	-13.7 (-102.1 to 36.8) *P *= 0.031	-12.8 (-165.2 to 108.5) *P *= 0.689	*P *= 0.169	*P *= 0.339

Pain mm	70.5 (7 to 98)	-0.5 (-40 to 25) *P *= 0.416	-1 (-39 to 53) *P *= 0.988	75 (10 to 92)	-7 (-27 to 35) *P *= 0.067	-4 (-34 to 29) *P *= 0.218	*P *= 0.356	*P *= 0.432

6MWT m	520 (381 to 660)	36.5 (-44 to 164.5) *P *< 0.001	29.3 (-150.5 to 145) *P *= 0.006	516 (428 to 656)	8 (-77 to 107) *P *= 0.183	-2 (-62.5 to 103) *P *= 0.520	*P *= 0.033	*P *= 0.049

IGF-1 ng/ml	4.3 (0 to 15.6)	-0,2 (-11.4 to 28.7) *P *= 0.300	-0.7 (-12.7 to 1.7) *P *= 0.019	3.3 (0 to 35.7)	-0.3 (-31.6 to 1.9) *P *= 0.148	-0.1 (-31.2 to 1.4) *P *= 0.109	*P *= 0.795	*P *= 0.570

IGFB3 ng/ml	1,449.2 (546.6 to 3,288.3)	-57.4 (-1,524.7 to 1,846.6) *P *= 0.977	291.1 (-1,383.3 to 2,821.8) *P *= 0.426	1,680.9 (269.5 to 3,274.2)	-33.5 (-1551.7 to 1,198.8) *P *= 0.881	242 (-1563 to 1,558.2) *P *= 0.796	*P *= 0.906	*P *= 0.872

^a^Wilcoxon signed rank test. ^b^Mann-Whitney *U *test. Median, range (min-max) for baseline, as well as median differences and *P *values at 15 and 30 weeks, are presented.

When calculated for the total group after 15 weeks of exercise, 6MWT was significantly improved (median, 25.1; *P *< 0.001), Table 3. Decrease of the pain threshold was significant (median, -9.6; *P *= 0.040), whereas the decrease of pain was not (median, -5.0; *P *= 0.052), Table 3.

**Table 3:** Clinical data for the study population (*n *= 49) at baseline, 15, and 30 weeks.

	Baseline median (range)	15 weeks median (range)	15-0 weeks difference median (range)	*P *value	30 weeks median (range)	30 to 0 weeks difference median (range)	*P *value
Pain threshold kPa	202.8 (105.9-380.5)	189.9 (112.2-305.3)	-9.6 (-102.1-75.6)	0.040	194.3 (107.6-472.3)	-8.8 (-165.2-108.5)	0.608

Pain 0 to 100	71 (7-98)	65.5 (3-99)	-5 (-40-35)	0.052	67 (14-95)	-2 (-39-53)	0.365

6MWT m	518 (381-660)	566 (418-689	25.1 (-77-164.5)	< 0.001	555 (367.5-661)	15.5 (-150.5-145)	0.009

IGF-1 ng/ml	4.2 (0-35.7)	3.0 (0-33.8)	-0.2 (-31.6-28.7)	0.085	2.7 (0-10.9)	-0.7 (-31.2-1.7)	0.004

IGFB3 ng/ml	1,549.4 (2,69.5-3,288.3)	1,634 (182.4-2,967.3)	-57.5 (-1,551.7-1,846.6)	0.889	1,795.4 (98.3-4,156.7)	272.9 (-1,563-2,821.8)	0.413

A significant association was found between 6MWT and the pain threshold at baseline (*r*^*s *^= 0.294; *P *= 0.040). Change in 6MWT (Δ6MWT) was correlated with changes in pain thresholds (ΔPT) after 15 weeks (*r*^*s *^= 0.339; *P *= 0.020) and after 30 weeks (*r*^*s *^= 0.407; *P *= 0.006).

### Influence of exercise on IGF-1

No significant between-group differences were found for changes in IGF-1 (*P *= 0.795) or the IGFBP-3 (*P *= 0.906) occurring during the 15-week low-intensity and high-intensity aerobic exercise, respectively.

In the NW group, IGF-1 did not significantly change during the 15-week exercise (*P *= 0.300), but it decreased at 30-week follow-up (*P *= 0.019). IGFBP-3 did not significantly change during the 15-weeks (*p *= 0.977) or during the 30 weeks (*P *= 0.426).

In the LIW group, IGF-1 did not significantly change during the 15 weeks (median, -0.340; *P *= 0.148), but it tended to decrease during the 30 weeks (*P *= 0.109). IGFBP-3 did not signficantly change during the 15 weeks (*P *= 0.881) or 30 weeks (*P *= 0.796).

When calculated for the total group, levels of either free IGF-1 or IGFB3 did not significantly change during the exercise period (Table 3).

### Effects on IGF-1 levels and pain

Basal IGF-1 did not correlate with baseline pain (*r^s ^*= -0.026; *P *= 0.861). To evaluate the relation of basal IGF to the change in pain, we did a partial correlation between baseline IGF and final pain and adjusted for baseline pain. We found a tendency to positive correlation (*r *= 0.278; *P *= 0.059). However, this positive tendency disappeared when age was accounted for (*r *= 0.245; *P *= 0.101).

Change in IGF-1 levels (ΔIGF) was associated with ΔPT at 15 weeks (*r *= 0.276; *P *= 0.058), and the correlation reached a significant level at 30-week follow-up (*r*^*s *^= 0.449; *P *= 0.005). No significant correlations were found between ΔIGF-1 and change in rating of pain (Δpain) when analyzed for the total group.

### IGF-1 level, pain, and CSF markers

A smaller group within the study population agreed to participate in the examination of cerebrospinal fluid at the start of the exercise period (*n *= 26). Their median age was 51 (46 to 55) years. At baseline, the number of tender points was 14.5 (12 to 16), pain threshold was 207 (161 to 258) kPa, pain level was 70 (44 to 83), and IGF-1 was 4.5 (2.2 to 9.3) ng/ml, implying that they did not significantly differ from the total sample.

Fifteen individuals were able to participate in a second examination of cerebrospinal fluid after 15 weeks. Their median age was 51 (34 to 59) years. At baseline, tender points for these 15 individuals was 15.0 (12 to 18), the pain threshold was 207 (177 to 255) kPa, the pain level was 70 (51 to 82), and IGF-1 was 4.2 (2.2 to 9.2) ng/ml, implying that they did not significantly differ from the total sample.

Changes in serum IGF-1 levels correlated positively with ΔCSF NPY (*r*^*s *^= 0.802; *P *= 0.001). The correlation between IGF-1 and ΔCSF SP tended to be significant (*r*^*s *^= 0.495; *P *= 0.072), Table 4.

**Table 4:** Correlation of changes in IGF-1, pain, and pain threshold, and changes in cerebrospinal markers

	ΔIGF-1 (serum)	ΔPain threshold	ΔPain
	0.495	0.539	0.075
ΔCSF SP	*P *= 0.072	*P *= 0.038	*P *= 0.790
	*n *= 14	*n *= 15	*n *= 15

	-0.164	0.335	0.016
ΔCSF NGF	*P *= 0.558	*P *= 0.204	*P *= 0.953
	*n *= 15	*n *= 16	*n *= 16

	0.802	0.288	0.315
ΔCSF NPY	*P *= 0.001	*P *= 0.318	*P *= 0.273
	*n *= 13	*n *= 14	*n *= 14

	-0.165	0.225	-0.119
ΔCSF IL-6	*P *= 0.573	*P *= 0.420	*P *= 0.673
	*n *= 15	*n *= 15	*n *= 15

	-0.164	-0.109	0.103
ΔCSF IL-8	*P *= 0.558	*P *= 0.688	*P *= 0.704
	*n *= 15	*n *= 16	*n *= 16

	-0.156	-0.579	0.329
ΔCSF MMP3	*P *= 0.594	*P *= 0.024	*P *= 0.231
	*n *= 14	*n *= 15	*n *= 15

Median and range (min-max) indicated. Spearmans rho(*r^s^*), two-tailed significance (*P*), and number of subjects (*n*) are presented.

Changes in pain threshold (ΔPT) were associated with ΔCSF SP (*r*^*s *^= 0.600; *P *= 0.023), and inversely with ΔCSF MMP-3 (*r*^*s *^= -0.569; *P *= 0.034), Table 4.

### Relations between CSF markers

Baseline CSF IL-6 was positively correlated with CSF SP (*r*^*s *^= 0.438; *P *= 0.032) and CSF MMP3 (*r*^*s *^= 0.409; *P *= 0.047). Furthermore, baseline CSF IL-6 was inversely correlated with CSF NGF (*r*^*s *^= -0.410; *P *= 0.042; *n *= 25) and CSF NPY (*r*^*s *^= -0.399; *P *= 0.053; *n *= 24).

### Type 1 error

Analyses of within-group analyses and between-group analyses comprised 40 analyses (Tables 2 and 3), and the upper level of of number of false signifcances was 1.58, which means that two significances might be false. Analyses of correlations between IGF-1, pain, and CSF markers (Table 4) comprised a total of 18 analyses, and the upper level of the number of false significances was 0.79, which means that one of the significances might be false.

## Discussion

The levels of bioactive IGF-1 and IGFBP-3 did not significantly change between an intervention group exercising 15 weeks at moderate- to high-intensity Nordic Walking (NW) and an active control group engaging in low-intensity walking (LIW). Lack of changes in IGF-1 levels is in line with a previous study reporting failure to show increase in IGF-1 in patients with FM [10]. In an exercise study with patients with a chronic kidney disorder engaging in regular exercise, no change was reported in IGF-1 [40]. Disease-specific characteristics, such as impaired physical function and chronic pain, may be possible reasons for the failure to increase IGF-1 and IGFBP-3 in our study.

A tendency to correlation was noted between baseline IGF-1 and the decrease of pain in response to exercise. However, this positive tendency disappeared when age was accounted for, and population studies show that IGF-1 levels correlate inversely with age [41]. It might be of interest to study levels of IGF-1 and pain during exercise in a larger group of FM patients homogeneous in age [41].

The pain threshold was found to decrease in our study population during the 15 weeks of exercise. This implies that the patients exercised at a level that activated their peripheral pain receptors [42], both in the NW and low-intensity walking group. When calculated for the total group, the pain scores tended to decrease after the 15-week exercise period. Pain scoring is a global rating of pain during the past week, thus including pain during activities and global well-being, aspects that can be expected to improve by both types of exercise. These different aspects of pain may explain differences in the two assessments. Another interesting finding was that change in the pain threshold were found to correlate with change in IGF-1. This indicates that serum free IGF-1 is associated with changes in sensitivity to pain, which must be further studied. The findings of this study support previous studies indicating that a higher level of IGF-1 in FM reflects better health [5,6,43,44].

Previous studies have shown that SP is increased in cerebrospinal fluid (CSF) of patients with FM [16-18]. CSF SP levels are relatively stable over time in FM patients; however, an increase in CSF SP over time in FM patients has been associated with small increases in pain or tenderness [16]. In our material, change in CSF SP was positively associated with changes in pain threshold. The change of CSF SP levels during aerobic exercise has, to our best knowledge, not been previously studied in patients with FM, but it has previously been found that SP is involved mainly in initiation rather than maintenance of chronic pain [45]. Regular exercise has shown beneficial effects on brain function [46]. Exercise effects on brain function, including mobilization of BDNF, are dependent on IGF-1 and IGF-1 uptake into the brain [47]. A possible interpretation of our data is that exercise-induced peaks of IGF-1 combined with high chronic levels of CSF SP may lead to long-term adaptation to tolerate physical strain. Thus, IGF-1 may promote resilience to pain in the CNS. In experimental diabetic neuropathy, treatment with IGF-1 reversed neuronal hyperactivity in the spinal cord and in the periaqueductal gray matter and prevented behavioral signs of pain [48]. In RA and AS patients, early response to exercise led to increased IGF-1 levels and reduced pain [49]. Furthermore, daily growth hormone treatment in FM patients led to increased IGF-1 and reduced pain levels [50]. Future studies are needed to determine short-term changes of the IGF-1 in relation to exercise in patients with FM.

We studied MMP3 in CSF for its potential role in structural reorganization and inflammation and found that change in CSF MMP-3 correlated negatively with change in pain threshold, which supports the notion that MMP3 is modulated during exercise [33,51]. The correlation between CSF MMP-3 and CSF IL-6 at baseline in our material may represent MMP-3 release induced by inflammatory cytokines [34,52,53], or stimulation of local inflammatory cytokine production by MMP-3 [54]. This study is, to our best knowledge, the first publication reporting changes in these substances during a prospective exercise period.

## Conclusions

Levels of free IGF-1 and IGFB3 in serum did not change during 15 weeks of aerobic exercise in patients with FM. Baseline IGF-1 was associated with a decrease of pain in response to exercise, but the tendency disappeared when adjusted for age.

Changes in levels of IGF-1 correlated positively with changes in SP and NPY in CSF and with changes in pain threshold. It appears that higher level of IGF-1 indicates less pain during exercise in FM, which must be further studied.

## Abbreviations

CSF: cerebrospinal fluid; FIQ: Fibromyalgia Impact Questionnaire; FM: fibromyalgia syndrome; IGF-1: insulin-like growth factor-1; IGFBP3: insulin-like growth factor-binding protein-3; IL-6: interleukin-6; IL-8: interleukin-8; MMP-3: matrix metalloproteinase 3; NGF: nerve growth factor; NPY: neuropeptide Y; PT: pain threshold; 6MWT: Six-minute Walk Test; SP: substance P.

## Competing interests

The authors declare that they have no competing interests.

## Authors' contributions

JB participated in study design, acquisition, analysis, and interpretation of data; MD, in acquisition, analysis, and interpretation of data; ME, in acquisition, analysis, and interpretation of data; MBo, in acquisition, analysis. and interpretation of data. KM worked with study conception, study design, acquisition, analysis, and interpretation of data. All the authors were involved in the drafting of the article and revising it critically for important intellectual content. All the authors approved the final version of the article.
